# Antitumor Response and Immunomodulatory Effects of Sub-Microsecond Irreversible Electroporation and Its Combination with Calcium Electroporation

**DOI:** 10.3390/cancers11111763

**Published:** 2019-11-09

**Authors:** Vitalij Novickij, Robertas Čėsna, Emilija Perminaitė, Auksė Zinkevičienė, Dainius Characiejus, Jurij Novickij, Saulius Šatkauskas, Paulius Ruzgys, Irutė Girkontaitė

**Affiliations:** 1 Faculty of Electronics, Vilnius Gediminas Technical University, 03227 Vilnius, Lithuania; 2 Department of Immunology, State Research Institute Centre for Innovative Medicine, 08410 Vilnius, Lithuania; 3 Biophysical Research Group, Vytautas Magnus University, 44404 Kaunas, Lithuania

**Keywords:** electroporation, electrochemotherapy, immune response, in vivo, murine myeloma, pulsed electric fields

## Abstract

In this work, we have investigated the feasibility of sub-microsecond range irreversible electroporation (IRE) with and without calcium electroporation in vivo. As a model, BALB/C mice were used and bioluminescent SP2/0 myeloma tumor models were developed. Tumors were treated with two separate pulsed electric field (PEF) pulsing protocols PEF1: 12 kV/cm × 200 ns × 500 (0.006 J/pulse) and PEF2: 12 kV/cm × 500 ns × 500 (0.015 J/pulse), which were delivered with and without Ca^2+^ (168 mM) using parallel plate electrodes at a repetition frequency of 100 Hz. Both PEF1 and PEF2 treatments reduced tumor growth and prolonged the life span of the mice, however, the PEF2 protocol was more efficient. The delay in tumor renewal was the biggest when a combination of IRE with calcium electroporation was used, however, we did not obtain significant differences in the final mouse survival compared to PEF2 alone. Anti-tumor immune responses were also investigated after treatment with PEF2 and PEF2+Ca. In both cases the treated mice had enlarged spleens and increased spleen T cell numbers, lower percentages of suppressor cell subsets (conventional CD4^+^CD25^+^ Treg, CD4^+^CD25^−^DX5^+^ Tr1, CD8^+^DX5^+^, CD4^+^CD28^−^, CD8^+^CD28^−^), changed proportions of Tcm and Tef/Tem T cells in the spleen and increased amount of tumor cell specific antibodies in the sera. The treatment based on IRE was effective against primary tumors, destroyed the tumor microenvironment and induced an anti-tumor immune response, however, it was not sufficient for complete control of tumor metastasis.

## 1. Introduction

Currently, different variations of electroporation (irreversible electroporation (IRE) [1], electrochemotherapy (ECT) [2], calcium electroporation [3,4], etc.) are implemented in experimental studies and in clinical trials, while IRE and ECT have been well established clinical methods for more than two decades. IRE triggers tissue ablation due to an applied electric field (irreversible cell membrane permeabilization) [1,5], while ECT relies more on a chemotherapeutic agent (i.e., bleomycin, cisplatin) which is delivered at a cellular level during a predominantly reversible permeabilization process [6,7]. At the same time, calcium electroporation is a relatively new pulsed electric field(PEF) treatment modality based on the delivery of supraphysiologic doses of calcium via electroporation [8]. Such an intracellular overload of calcium induces acute cell death as a result of ATP depletion that leads to necrosis [9]. Calcium electroporation also seems to be more effective against cancer cells compared to normal cells, thus, introducing selectivity and efficiency comparable to that of electrochemotherapy [10]. The treatment has been successfully used for malignant melanoma treatment [10,11], however, it is not always effective with other tumors. For example, breast cancer progression with new cutaneous metastases outside the treatment area was detected after calcium electroporation treatment [10].

Prevention of metastases after PEF treatment seems to be one of its biggest challenges. Therefore, the immunomodulatory effects of electroporation have been studied and efforts to potentiate the immune response in combination with other methods made [12,13], i.e., electroporation can be used in combination with antibodies to programmed cell death protein 1 (anti-PD1) immune checkpoint blockade [12] or electrotransfer of plasmids coding cytokine genes like interleukin-12 (IL-12), TNF α, and interleukin-15 (IL-15) that stimulate anti-tumor immune responses [14,15]. Nevertheless, it was recently shown that IRE alone can induce significant immunomodulatory effects [16], while calcium electroporation can trigger long-lasting immunity and changes in cytokine patterns, hence enabling complete remission of tumors [17].

It is also agreed that electroporation is a safe treatment option to use in patients, however, it may still involve pain, muscle contractions and be not 100% effective in long-term follow-up [18]. Therefore, to minimize muscle contractions and to ensure a more uniform tumor exposure by PEF (due to impedance mitigation), the whole IRE/ECT field is slowly moving towards the shorter pulse range (i.e., nanosecond pulses—nsPEF) [19,20,21]. Shorter pulses allow a more selective treatment with formation of transient nanopores in both the plasma and organelle membranes, leading to an immediate increase of intracellular Ca^2+^, reactive oxygen species, DNA fragmentation and caspase activation, which is not always achievable in microsecond range [22,23]. Lastly, nanosecond pulses may trigger an immunogenic cell death [24].

Therefore, this work focuses on the combination of IRE and calcium electroporation for the possible potentiation of immunomodulatory effects of both treatments. We present the first in vivo experimental data showing the efficacy of sub-microsecond range IRE combined with calcium electroporation and corresponding immunomodulatory effects.

## 2. Results

Two PEF protocols (PEF1—12 kV/cm × 200 ns × 500; PEF2—12 kV/cm × 500 ns × 500) using parallel plate electrodes were tested on a SP2/0 myeloma tumor model. For this purpose, stable luciferase-expressing SP2/0 cells (named SP2/0 Luc) were established. Innoculation of such cells under the skin of BALB/c mice forms luciferase-expressing tumors, that become luminescent after injection of D-luciferin. The following mice groups were used in the study: (1) untreated tumor- bearing mice (CTRL); (2) tumor-bearing mice treated with CaCl_2_ (CTRL+Ca); (3) treated with PEF1; (4) treated with PEF2; (5) treated with PEF1 + CaCl_2_ (PEF1+Ca); (6) treated with PEF2 + CaCl_2_ (PEF2 + Ca)_._ PEF protocols of different energy (PEF1: 0.006 J/pulse; PEF2: 0.015 J/pulse) were selected to trigger different levels of tissue ablation for comparison purposes and possible potentiation by calcium electroporation.

### 2.1. Bioluminescence Assay

**Figure 1:**
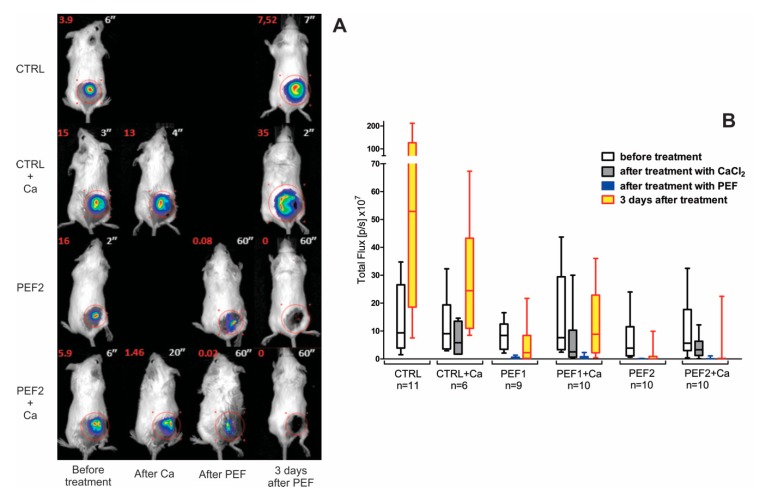
The changes of bioluminescence of the tumors after pulsed electric fields (PEF) and calcium electroporation (PEF + Ca), where (**A**) representative pictures of bioluminescence in vivo; total fluxes (p/s × 10^7^) are shown in red on the left upper corner and the exposure times (in seconds) are shown on the right upper corner of every image; (**B**) changes in tumor bioluminescence immediately after the treatment and three days later; CTRL—tumor-bearing mice ; CTRL + Ca—tumor-bearing mice treated with CaCl_2_; PEF1—12 kV/cm × 200 ns × 500; PEF2—12 kV/cm × 500 ns × 500.

Bioluminescence analysis was performed to assess the treatment efficacy immediately after the pulse and three days post-electroporation, which is not always possible using volumetric analysis due to scab and necrotic tissue formation. The results are summarized in Figure 1. As it can be seen in the treatment-representative images (Figure 1A), after PEF there is a residual luminescence signal, indicating that not all the cells are killed instantly, whereas a complete response is achieved when checked after 3 days. Injection of calcium does reduce the luminescence signal, however, this is a transient process and without PEF the tumor continues to grow in a longer follow-up. Both selected PEF protocols are applicable for tissue ablation without calcium, which is confirmed in Figure 1B. The luminescence of tumors is reduced by several factors, and the effect is comparable immediately after treatment for both pulsing protocols. However, 3 days post-treatment the differences are intensified and on average the PEF1 treatment triggers a weaker tumor response. Surprisingly, the application of calcium increases the deviation of the results and triggers a weaker response (compared to PEF) in the first days of the treatment. This phenomenon is non-detectable using conventional volumetric analysis techniques due to the formation of scabs.

### 2.2. Scab Formation

**Figure 2:**
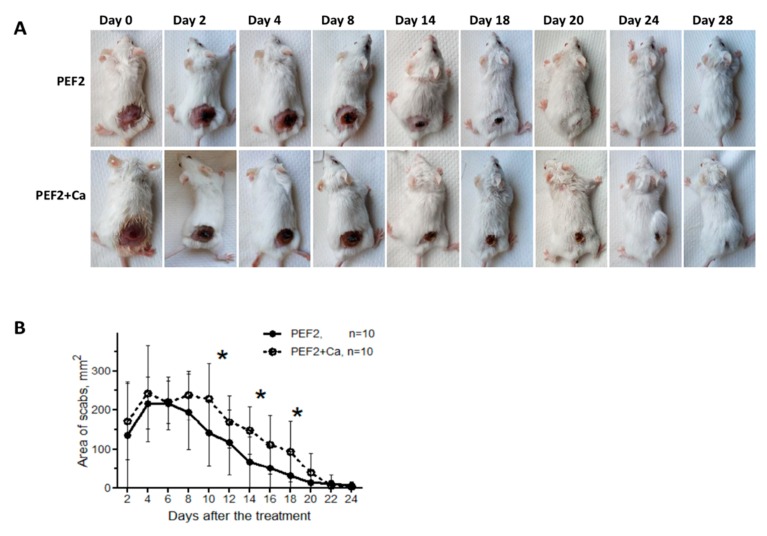
The changes in scab area depending on the treatment, where (**A**) representative images post-treatment; (**B**) dynamics of scab area; PEF2 protocol—12 kV/cm × 500 ns × 500 treatment was used separately and in combination with CaCl_2_. Asterisk (*) indicates significant differences according to Mann Whitney test (*p* < 0.005) between the mice groups.

Scab formation is a typical consequence of PEF-based treatments. During the first days post-treatment it prevents accurate volumetric estimation of the tumor size. However, when the tumors are of comparable size it may serve as a supplementary indicator of the treatment intensity. We have compared the changes in scab area during PEF2 treatment with and without calcium electroporation. The results are summarized in Figure 2.

As it can be seen, on average the scab area after PEF + Ca treatment was higher compared to PEF treatment alone, indicating higher ablation. The results of the luminescence assay (Figure 1B) provide evidence that it was not the case for the tumor, which implies that during calcium electroporation the energy losses in the skin are higher and as a result, less energy is absorbed by the tumor and a weaker cancer ablation is triggered (refer to Figure 1B).

### 2.3. Volumetric Tumor Changes and Survival

Further, we analyzed the volumetric changes of tumors every two days after the treatment. The selected cancer model is metastatic to lymph nodes. Therefore, we calculated and presented separately the volumes of the primary tumors (T) and the sum of volumes of the primary tumor and lymph nodes enlarged due to metastasis (T + LN). Tumors were measured until the primary tumor reached about 3000 mm^3^ (according to the acquired bioethics approval).

**Figure 3:**
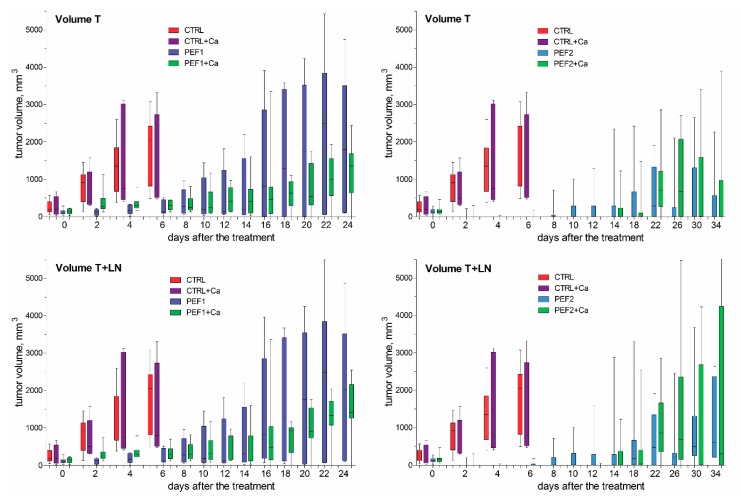
Volumetric changes of the tumors after pulsed electric fields (PEF) and calcium electroporation (PEF + Ca) treatment. Volumes of the primary tumor (Volume T) and the sum of the volumes of primary tumor + secondary tumors in lymph nodes (Volume T + LN) are shown. CTRL—tumor bearing control mice without treatment; CTRL+Ca—tumor bearing mice treated with CaCl_2_; PEF1 and PEF2—tumor bearing mice treated with PEF1 protocol: 12 kV/cm × 200 ns × 500 (0.006 J/pulse) or PEF2 protocol—12 kV/cm × 500 ns × 500. PEF1+Ca and PEF2+Ca—tumor-bearing mice treated with PEF and CaCl_2_. Primary tumors in CTRL and CTRL+Ca cases developed rapidly, thus the influence of metastases in LN is non-present.

As it can be seen in Figure 3, the dynamics of the tumor growth are significantly altered by electroporation. In a long-term, calcium by itself does not inhibit tumor growth and the response is similar to untreated tumor-bearing control. The PEF1 protocol induced a significant delay in tumor growth, however a complete response was not achievable. On the other hand, a definitive potentiation of the PEF1 treatment by calcium electroporation was observed. In case of PEF2 protocols the tumor growth delay was even more apparent. However, on a longer scale (more than 20 days), no significant differences (Mann Whitney test, *p* < 0.005) were observed between PEF1/PEF1 + Ca, PEF2/PEF2 + Ca treatments.

**Figure 4:**
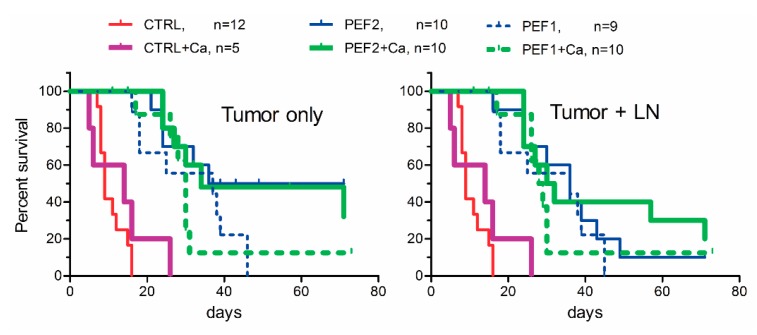
Kaplan-Meier survival curves of mice with SP2/0-luc tumors treated with pulsed electric field (PEF) or PEF + Ca. The endpoint in survival curves was taken at the time when the volume of the primary tumor (**left**) or the sum of primary tumor and enlarged lymph nodes (LN) due to metastasis (**right**) reached 3000 mm^3^. CTRL (not treated tumor bearing mice), CTRL + Ca (mice treated with calcium alone) PEF1 (12 kV/cm × 200 ns × 500); PEF2 (12 kV/cm × 500 ns × 500), PEF1 + Ca and PEF2 + Ca—mice treated with CaCl_2_ prior to treatment with PEF1 or PEF2.

Significant differences (*p* < 0.005) were detected between the mice groups CTRL/PEF1, CTRL/PEF2, CTRL + Ca/PEF 1+ Ca, CTRL + Ca/PEF2 + Ca (*p* < 0.005) at days 2, 4 and 6. Further, we have analyzed the survival of the mice with tumors. The results are summarized in Figure 4. Significant differences in median survival between CTRL and PEF1, PEF2, PEF1+Ca, PEF2 + Ca-treated groups, and also between CTRL + Ca, PEF1 + Ca and PEF2 + Ca-treated mice were detected (*p* < 0.0006 according Log-rank Mantel-Cox and Gehan-Breslow-Wilcoxon tests). PEF2 separately and in combination with calcium produced the most successful treatment outcome. When the endpoint was estimated according the size of the primary tumor (Figure 4, left), the responses were identical for both treatments (PEF2 and PEF2 + Ca).

However, when the endpoint was estimated according to the size of primary tumor + metastasis a delay between calcium and calcium-free treatments was observed. It was not the case for PEF1 + Ca treatment when the weaker tumor ablation (both due to lower energy protocol and losses on skin due to injection of calcium) was triggered. The delay was apparent during the initial stages of tumor renewal (refer to Figure 3), however, on a longer scale the potentiation of the treatment by calcium electroporation was diminished. A summary of the experimental outcomes is presented in Table 1.

**Figure 5:**
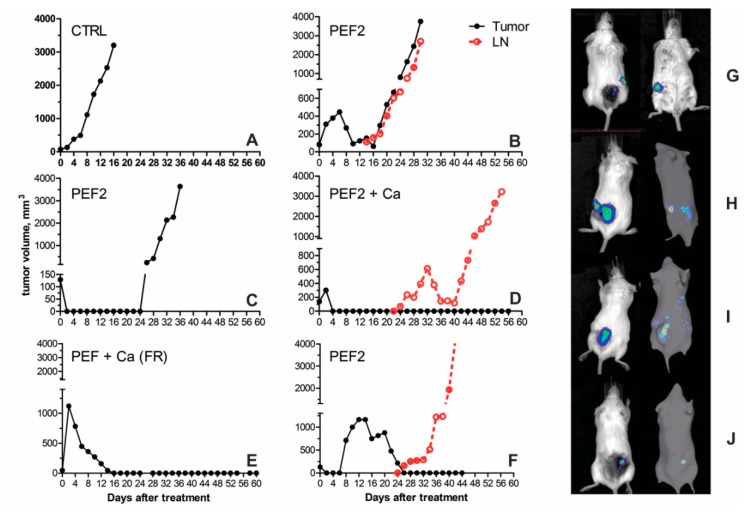
Examples of dynamics of tumor and metastases development in SP2/0 Luc myeloma tumors bearing mice with and without pulsed electric field (PEF) treatment, where (**A**–**F**) mice cases to highlight the heterogeneity in response to the treatments; (**G**–**J**) exemplary 2D and 3D images of metastases development and/or primary tumor regrowth.

As it can be seen in Table 1, 20% of mice fully recovered after PEF2 + Ca treatment. A diminished occurrence of metastases in lymph nodes tendency was also observed (40% versus 70% PEF2 only). This was not the case for the PEF1 protocol. Even though there was a fully recovered animal in the PEF1 + Ca group, however, metastases occurred more frequently when calcium was used, which we believe is a consequence of higher energy losses on the skin and thus, weaker tissue ablation. No animals survived when PEF1 or calcium only treatments were used. In case of tumor-bearing untreated controls (CTRL) the smaller number of metastases (23.4%) was influenced by the short lifespan of the mice with untreated tumors since the primary tumors reached 3000 mm^3^ before the typical occurrence of metastases, which develop at a later stage. Some exemplary observed dynamics of metastases development are shown in Figure 5.

The general tendency is that after the PEF treatment, the primary tumor growth or renewal was significantly hindered or delayed (Figure 5C,D,E) compared to untreated control (Figure 5A). The metastases appear at a later stage (Figure 5B,D,F) and primarily in the lymph nodes (Figure 5G,H), while multiple localizations are also possible (Figure 5I). The renewal of the primary tumor after what it seemed to be a successful treatment was also apparent in some cases (Figure 5C,J). Taking into account the multifactorial mechanism of action of calcium electroporation and IRE we have further analyzed the changes in lymphocyte subsets and immunological responses triggered by the more successful PEF2 treatment.

### 2.4. Lymphocyte Subsets in Spleen, Lymph Nodes and Tumors 

**Figure 6:**
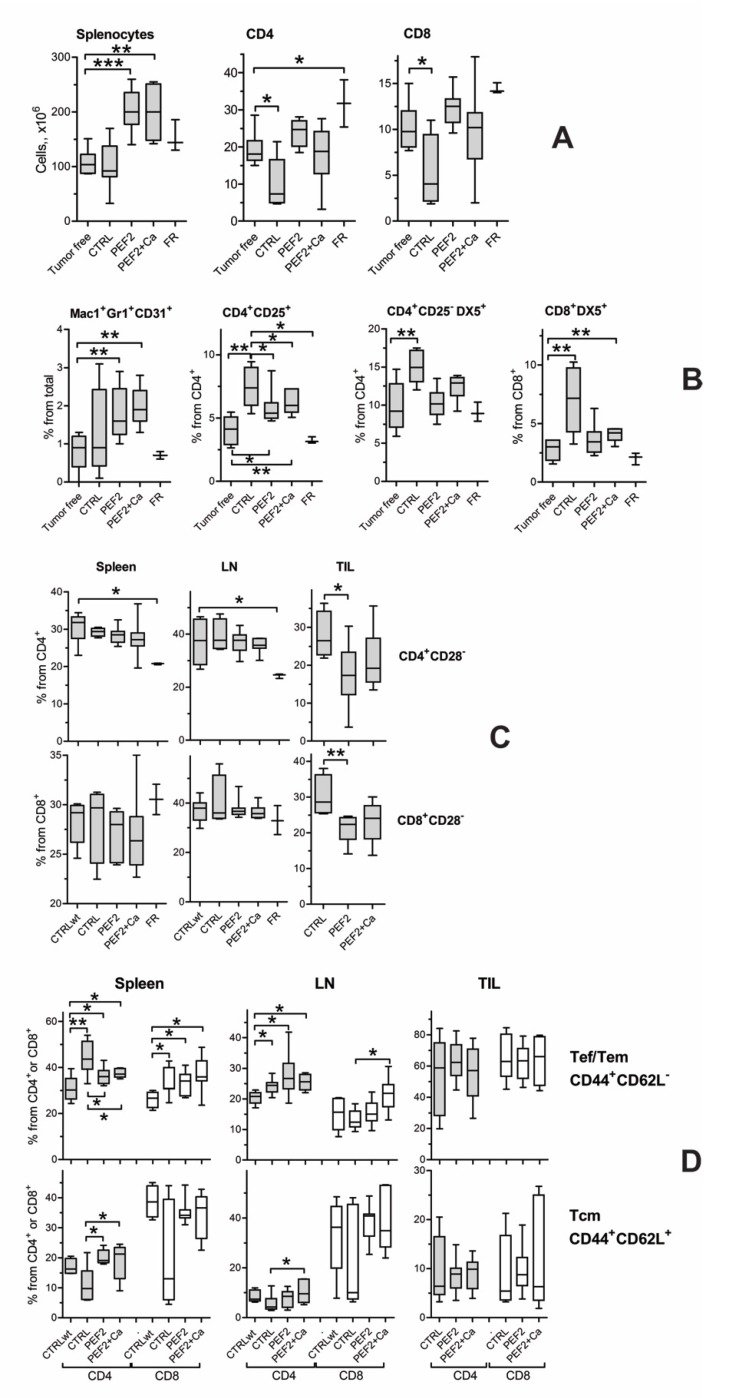
Lymphocyte subsets, where (**A**) the total numbers of splenocytes, CD4 and CD8 T cells in spleens; (**B**) percentages of myeloid suppressor Mac1^+^Gr1^+^CD31^+^, CD4^+^CD25^+^ and CD4^+^CD25^−^Dx5^+^ (Tr1) suppressor T cells, CD8^+^DX5^+^ T cells in the spleens; (**C**) percentages of CD28-negative CD4 and CD8 T cells; (**D**) percentages of Tef/Tem (T effector memory cells) and Tcm (T central memory) cells. LN—lymph nodes, TIL—tumor infiltrated lymphocytes. Significant differences between the mice groups according to Mann Whitney test are marked by asterisks: * *p* < 0.05, ** *p* < 0.005, *** *p* < 0.0005. Tumor-free mice (*n* = 5–7), CRTL-tumor-bearing untreated or only CaCl_2_-treated mice (*n* = 4–8), PEF2- treated mice (*n* = 9), PEF2 and CaCl_2_-treated mice (*n* = 7), FR-fully recovered mice (*n* = 3).

The mice were sacrificed when the tumor volume reached 3000 mm^3^. Spleens, lymph nodes (LN) and tumors were removed and the subsets of immune cells were analyzed by flow cytometry. The cells from fully recovered mice were analyzed at 72 days after the treatment. The control cells from tumor free age-matched mice were included in the analysis. The spleens from PEF2 and PEF2 + Ca-treated mice were enlarged and the numbers of splenocytes (on average 200 × 10^6^) were significantly (about two times) increased compared to tumor-free (average 107 × 10^6^) and tumor-bearing controls (average 100 × 10^6^) (Figure 6A). Tumor-bearing controls had significantly less CD4 (10.3 ± 6.5 × 10^6^) and CD8 CD4 (5.4 ± 3.7 × 10^6^) T cells in spleens compared to tumor-free mice (CD4 19.3 ± 4.8 × 10^6^ and CD8 10.2 ± 2.6 × 10^6^), while PEF2 and PEF2+Ca treatment restored the numbers of T cells in the spleens up to the tumor-free mice level (CD4 23.7 ± 3.5 × 10^6^ after PEF2 treatment and 18.2 ± 8.2 × 10^6^ after PEF2 + Ca treatment; CD8 12.3 ± 1.9 × 10^6^ after PEF2 treatment and 10 ± 4.9 × 10^6^ after PEF2+Ca treatment). The numbers of splenocytes and T cells in the spleens of fully recovered mice were higher than in spleens of control mice, however due to low number (*n* = 3) of fully recovered mice, the differences were not statistically significant. The total numbers of cells in lymph nodes and TIL (tumor infiltrated lymphocytes) were not analyzed. The CD4/CD8 ratios in spleen (2.0 ± 0.3), LN (2.4 ± 0.3) and TIL (0.51 ± 0.36) were similar in mice from all groups.

Further, we analyzed the number of myeloid precursors (CD11b/Mac1^+^Gr1^+^CD31^+^), CD4 suppressors (CD4^+^CD25^+^), Tr1 suppressors (CD4^+^CD25^−^Dx5^+^) and CD8^+^Dx5^+^ cells in spleens. The percentages of all subsets were significantly increased in the spleens of tumor bearing controls compared to the spleens from tumor free mice (Figure 6B).

Electroporation did not influence the number of myeloid cells, however, the percentages of myeloid cells in the spleens of fully recovered mice were at the same level as in the spleens from tumor-free mice. The average percentages of CD4^+^CD25^−^Dx5^+^ cells in tumor-free, in tumor-bearing PEF2 and PEF2 + Ca-treated mice were 9.5%, 15%, 10.2% and 12.4%, respectively. The average percentages of CD8^+^Dx5^+^ cells in tumor-free, in tumor-bearing PEF2 and PEF2+Ca-treated and fully recovered mice were 2.7%, 6.9%, 3.6% and 4%, respectively. The levels of CD4^+^CD25^+^ cells in PEF2 and PEF2 + Ca-treated mice were lower than in tumor-bearing controls, but higher than in tumor- free mice (average of 4% in tumor-free mice, 6.9% in tumor-bearing mice and around 4% in PEF2 and PEF2 + Ca-treated mice).

The amount of all mentioned subsets of T cells in spleens of fully recovered mice was similar to tumor-free controls (Figure 6B). The percentage of CD28 negative cells among CD4 and CD8 T cells were analyzed in spleens, LN and TIL. The percentages of CD28 negative cells were lower between CD4^+^ (but not CD8^+^) T cells from spleens and LN of fully recovered mice compared to tumor-free mice (Figure 6C). The level of CD28 negative CD4 and CD8 T cells in spleen and LN did not differ between tumor free control, tumor-bearing control, PEF2 and PEF2+Ca-treated mice. However, the levels of CD4^+^CD28^−^ and CD8^+^CD28^−^ cells were significantly lower among TIL in PEF2 and PEF2+Ca-treated mice (average 17–23%) compared to tumor-bearing controls (about 30%) (Figure 6C). PEF2 and PEF2+Ca treatment did not have influence on the percentage of CTLA-4 expression on CD4^+^ and CD8^+^ splenocytes (Figure S1).

The proportion of T effector memory (Tef/Tem) and T central memory (Tcm) cells between CD4 and CD8 T cells were analyzed in spleens, LN and TIL (Figure 6D). The percentage of these cells in TIL did not differ in any of the mice groups, meaning that PEF2 treatment did not influence the levels of Tef/Tem and Tcm in TIL. Tumor growth evoked an increase of CD4 and CD8 Tef/Tem cells in the spleen, CD4 Tef/Tem in LN, but a decrease of the portion of CD4 and CD8 Tcm cells in spleen and LN. PEF2 (with and without Ca) treatment caused normalization of the levels of CD4 Tef/Tem cells in spleen, CD4 Tcm in spleen and LN, i.e., the percentage of mentioned cell subsets in treated mice was similar to that observed in tumor-free mice.

We have also investigated the expression of CD28 on Tem/Tef and Tcm T cells (Table S1). There is a tendency towards an increased percentage of CD28^high^ cells among CD4 Tem/Teff in spleen, lymph nodes and TIL after treatment with PEF2 and PEF2 + Ca. Similarly, increased rates of CD4 Tcm in lymph nodes and TIL and CD8 Tem/Teff/Tcm in TIL were observed after the PEF2 and PEF2 + Ca treatments (compared to tumor-free and tumor-bearing untreated control). CD28 is an important receptor for T cell activation and higher expression of CD28 on T cells of treated mice shows better activation ability. In summary, tumor treatment with PEF2 (with or without CaCl_2_) caused the same effects on the composition of lymphocytes in spleen, LN and TIL.

### 2.5. Immune Response

**Figure 7:**
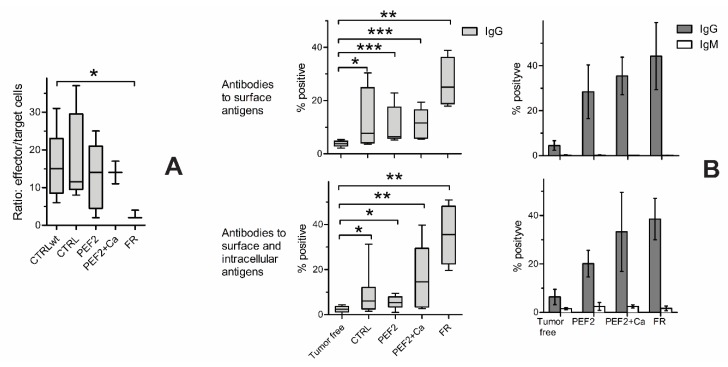
Anti-tumor immune response, where (**A**) Cytotoxic T Lymphocyte (CTL) killing assay. Y axis: Ratio effector/target cells when 50% of tumor cells are killed. Tumor free mice (*n* = 5), CTRL-tumor bearing mice (*n* = 6), PEF2 protocol treated mice (*n* = 5), PEF2 + Ca treated mice (*n* = 2), FR-full recovered mice (*n* = 3); (**B**) Antibodies to surface and surface + intracellular SP2/0 myeloma cell antigens in murine sera. Immunoglobulin G (IgG) antibodies were determined in sera of tumor-free mice (*n* = 8), CTRL (*n* = 8), PEF2 (*n* = 8), PEF2 + Ca (*n* = 7), FR (*n* = 4) (on the left). IgM and IgG antibodies (on the right) were determined in four selected samples of IgG anti-SP2/0 positive sera from every mouse group. Significant differences according to Mann Whitney test between the mice groups are marked by asterisks: * *p* < 0.05, ** *p* < 0.005, *** *p* < 0.0005.

PEF2 and PEF2 + Ca-induced anti-tumor immune response was evaluated using CTL-mediated killing assay and the amount of anti-tumor specific antibodies in serum. The ability of CTL to kill tumor cells was estimated as a ratio of effector/target cells when luminescence of tumor cells decreased 50%. CTL generated from the spleens of tumor free, tumor bearing control mice, PEF2 and PEF2 + Ca treated mice had similar ability to kill SP2/0 Luc tumor cells (Figure 7A). The ratios of effector/target cells were on average about 12–18. But CTL obtained from fully recovered mice had much better ability to kill the tumor cells. The ratio of effector/target cells was about 3.

Next, we investigated whether the PEF2 and PEF2 + Ca treatment can affect the generation of SP2/0 tumor cells specific antibodies in the serum. We determined antibodies to surface and intracellular antigens (Figure 7B). Different dilutions of the sera were tested. The results with 1:100 and 1:2000 serum dilutions for determination of antibodies to surface and intracellular antigens respectively are presented in Figure 7B. Such dilutions were chosen since the results show differences in levels of antibodies between separate mice groups. The amount of antibodies was estimated according to the percentage of fluorescence of antibody-bound tumor cells. The percentages of positive cells were set up as negative control (cells stained with secondary antibodies only).

Tumor-bearing mice already had antibodies to tumor cells surface and intracellular antigens when compared to tumor-free mice. The levels of antibodies (according to the average levels) increased in PEF2 + Ca-treated mice and especially in fully recovered mice. Four sera with the highest amount of antibodies were taken from every mice group for determination of IgM and IgG specific antibodies. The results in Figure 7 show that all tumor cell specific antibodies are IgG class.

## 3. Discussion

In this work, sub-microsecond range irreversible electroporation was used separately and in combination with calcium electroporation to treat SP2/0 Luc myeloma tumors. It was shown that application of IRE (12 kV/cm, 200/500 ns bursts) can be used for primary tumor ablation. However, in many cases the renewal of the primary tumor or the metastases was observed. Indeed, in order to achieve higher efficiency of tissue ablation (can be accompanied even with thermal effects [25]), higher PEF amplitudes (20–40 kV/cm) can be used [23,26,27], however, improvement of the IRE-induced systemic immune response could be an alternative solution. Calcium electroporation is known to trigger an immune response [17], therefore, improvement of IRE with the processes that are observed in calcium electroporation for prevention of metastasis was desired and was analyzed in this study. Two protocols with linear increase of burst energy (0.006 and 0.015 J/pulse, proportional to pulse duration) were used to ease the comparison in terms of future protocol design.

Cytotoxic effects of calcium electroporation, which are comparable with conventional ECT procedures, are frequently reported [10]. In our study, cytotoxic effects of calcium were also observed, however, mainly as a delay in tumor growth when compared to PEF treatment alone (first 25 days of the experiments). The result is in agreement with calcium effects reported in in vivo or clinical studies [3,11]. However, on a longer scale (more than 25 days) the differences were minor compared to PEF only treatment. The result could be justified by the usage of the different tumor cell models in the in vivo experiments. However, we believe that the primary reason for lack of long-term additive effects between 200 ns IRE and calcium electroporation is the conductivity gradients, which are increased during injection of a highly conductive calcium suspension. It is known that conductivity differences can influence electroporation [28,29], also part of the tumor might have been reversibly permeabilized, due to a dissipation of pulse energy on the skin. The observed tumor bioluminescence and scab area data support this hypothesis (refer to Figure 1B and Figure 2). Moreover, when calcium electroporation was combined with 500 ns IRE pulses (higher intensity), the treatment effects were more profound (i.e., the tumor growth delay increased and the frequency of metastases decreased), which does not contradict the hypothesis.

We also investigated the influence of nsPEF IRE and its combination with calcium electroporation on the numbers of different subsets of suppressor cells, formation of T cell memory response, cytotoxic activity to tumor cells and development of anti-tumor specific antibodies. Like Guo et al. [27] we found that the PEF-based treatment induced splenomegaly and increased the number of spleen T cells. The effects were observed for both the PEF2 only and PEF2 + Ca treatments. Suppressor cells play an important role in tumor immunology since they inhibit anti-tumor immune responses and promote the growth of the tumors. Until now, the effect of electroporation was investigated in the context of conventional Treg (CD4^+^CD25^+^ FoxP3^+^) and myeloid-derived suppressor cells (MDSC) (Mac1^+^Gr1^+^). For example, it was shown that tumor growth induces development of conventional Treg and MDSC [27,30], while IRE, ECT and calcium electroporation decrease the number of conventional Tregs and MDSC [11,26,27]. Our results are in agreement with the finding that the numbers of conventional Treg (CD4^+^CD25^+^) cells were increased in spleens of tumor-bearing mice, while electroporation reduced the number of these cells to a level comparable with tumor-free mice. Mac1+Gr1+ MDSCs are heterogeneous cell populations of myeloid cells [31], therefore, we investigated the amount of immature myeloid precursors Mac1^+^Gr1^+^CD31^+^ that are responsible for suppression of cytotoxic activity of CD8 T cells [32]. As a result, we have observed increased amounts of these cells in the spleens of tumor-bearing mice and PEF2 treatment did not reduce the numbers of Mac1^+^Gr1^+^CD31^+^ cells in the spleens.

Additionally to the conventional Treg and MDSC cells, other suppressor cell subsets are known to play an important role in tumor immunology. There are CD4^+^ type 1 T regulatory (Tr1) cells, CD8^+^DX5^+^, CD8^+^CD28^−^, CD4^+^CD28^−^. Tr1 (CD4^+^CD25^−^DX5^+^LAG-3+FoxP3^−^ regulatory T (Tr1) cells that secrete high amounts of interleukin-10 (IL-10) and transforming growth factor beta (TGF-β). IL-10 and TGF-β reduce synthesis of interferon-gamma (IFN-γ) and TNF-α by pro-inflammatory CD4 T cells, thus, lower tumor-specific cytotoxicity of CD8 cells, inhibit the main functions of dendritic cells and NK cells and this way induce tolerance to tumor cells [33,34]. The role of CD8^+^DX5^+^ cells in tumor immunology is not completely clear. It was shown that these cells have potent NK-like cytotoxic activity against multiple tumor targets [35], however, other data demonstrates that CD8^+^DX5^+^ cells regulate the immune response by killing antigen-bearing dendritic cells and in this way suppress T-cell responses [36]. Some data show that CD8^+^DX5^+^ cells are associated with an activation/memory phenotype and are biased towards apoptosis [37].

CD28^+^ T cells lose CD28 expression after repeated antigen stimulation. CD28^−^ T cells accumulate with aging and during chronic inflammatory diseases, immunodeficiency, and specific infectious diseases [38]. A noticeable expansion of CD4^+^CD28^−^ subsets was found in patients with invasive cervical carcinoma and it is believed they have immunosuppressive properties [39]. CD8^+^CD28^−^ T cells are a subpopulation of regulatory T cells that inhibit CD4 cell activation, proliferation, secretion of pro-inflammatory cytokines. Infiltration of CD8^+^CD28^−^ T cells in tumor microenvironments and increased numbers in the circulation of cancer patients are associated with poorer prognoses [40,41].

Here, for the first time the influence of tumor electroporation on the amount of the mentioned cell subsets was analyzed. We have shown that the amount of Tr1 (CD4^+^CD25^−^DX5^+^) and CD8^+^DX5^+^ increase in the spleens of tumor–bearing mice when compared with tumor-free mice (cells similar to conventional Treg (CD4^+^CD25^+^)). The numbers are reduced in the spleens of mice where tumors were treated with electroporation. We demonstrated that the amount of CD4^+^CD28^−^ cells is lower in fully recovered mice in comparison with tumor-free and tumor-bearing mice. Most importantly, we found that the percentage of CD4^+^CD28^−^ and CD8^+^CD28^−^ cells among tumor-infiltrated CD4 and CD8, respectively, were significantly lower in PEF-treated tumors in comparison with infiltrated CD4 and CD8 T cells in untreated tumors. These data indicate that electroporation can destroy the tumor microenvironment and trigger an antitumor immune response by reducing the numbers of suppressor cells.

Previous studies showed that electroporation induce memory T cell development [27]. Our data also confirm that electroporation of the tumors can modify the proportions of Tef/Tem and Tcm CD4 and CD8 T cells in spleen and lymph nodes, but not between tumor-infiltrated lymphocytes.

We have also investigated if electroporation of the tumors induces an anti-tumor immune response. CTL are very important for the antitumor immune response since they can kill tumor cells. CTL obtained only from fully recovered mice had increased cytotoxic activity to tumor cells, however the CTL obtained from untreated or PEF2/PEF2 + Ca-treated tumor-bearing mice did not have increased cytotoxic activity. However, we have determined the development of tumor-specific antibodies in the serum of mice treated by PEF. It is known that autoantibodies to tumor-associated antigens are detected in the sera of cancer patients [42]. Autoantibodies to surface and intracellular antigens of SP2/0 tumor cells were obtained in tumor-bearing mice. The amounts of those antibodies were increased in PEF2 + Ca-treated tumor-bearing mice and especially in fully recovered mice. IgG class specific antibodies indicate participating of CD4 T cells in anti-tumor immune response.

Nevertheless, the induced anti-tumor immune response was not sufficient for complete control of tumor metastasis. We believe that in order to optimize the methodology and improve the efficacy, the influence of calcium concentration should be examined in future works. We have used a typical value of 168 mM for better comparison with available studies, however, the immunogenicity with PEF may scale with calcium concentration. We have also shown that injection of calcium introduces deviation in the tumor response, which we believe is the effect of conductivity gradients. Further confirmation of the hypothesis is required, which may result in the development of higher efficiency PEF protocols when taken into account. Also, one of the solutions could be application of invasive needle-type electrodes arrays to minimize the energy losses on the skin. Lastly, we have used a single treatment, however, application of a series of treatments during the first week may increase the number of complete responses. Combination with other treatments is always an option.

## 4. Materials and Methods

### 4.1. Generation of Luciferase Expressing Sp2/0 Myeloma Cells

BALB/c mouse myeloma SP2/0 cells were maintained in RPMI 1640 supplemented with 2 mM glutamine, 100 U/mL penicillin, 100 mg/mL streptomycin and 10% of fetal calf serum (FCS). All cell culture reagents were obtained from Gibco (Thermo Fisher Scientific, Grand Island, NY, USA). The cells were cultured at 37 °C, 5% CO_2_. SP2/0 cells were electro-transfected (4 × 100 µs × 1.2 kV/cm) with Luciferase-pcDNA3 plasmid (Adgene plasmid #18964, a kind gift from William Kaelin, Harvard Medical School, Boston, MA, USA)) [43] linearized with Bgl II. The transfected cells were selected with 400 µg/mL of G418 Sulphate (Carl Roth GmbH, Karlsruhe, Germany) and the surviving cells were cloned in 96-well plates by limiting dilution. Cells from the wells with single growing clones were tested for expression of luciferase using an in vitro bioluminescent assay. For this purpose half of the cells were transferred to white plates with 96 wells. D-Luciferin (Promega, Madison, WI, USA) was added to the cells to a final concentration of 150 µg/mL. The luminescence of SP2/0 cells was evaluated using a Synergy 2 microplate reader and Gen5 software (BioTek, Winooski, VT, USA). The total luminescence was measured every 10 min for 4 h, at 37 °C. The cell clones were compared and selected according to the maximal luminescence (in RLU) over all kinetic read. Luciferase-expressing cells were grow up, frozen in medium containing 90% of fetal calf serum (FCS) and 10% of DMSO and stored in liquid nitrogen until used. The established luciferase- expressing cell lines were named SP2/0 Luc.

### 4.2. Mice and Tumor Induction

BALB/c mice were bred and housed in the mouse facility of the State Research Institute Centre for Innovative Medicine (Vilnius, Lithuania). 1 × 10^6^ of SP2/0 Luc myeloma cells in phosphate-buffered saline (PBS) were inoculated under the skin on the back of 6–8 week old mice. The tumors were allowed to establish and grown until they reached 4–10 mm in diameter and were ready to treat. The sizes of the tumors were evaluated based on volumetric measurements and according to the intensity of tumor luminescence prior to and every 2–3 days after the treatment. The mice with tumors were sacrificed by cervical dislocation when the primary tumor volumes reached about 3000 mm^3^. The full recovered mice were kept up to 72 days after the treatment. Spleens, lymph nodes and tumors were removed and used for flow cytometry and cytotoxic killing assay. Blood was taken out from the heart of killed mice and the serum used for determination of anti-tumor specific antibodies. All experimental protocols were approved by the Lithuanian State Food and Veterinary Service (2018-04-approval no. 02-24) and the study was carried out in strict accordance with the recommendations in the Guide for the Care and Use of Laboratory Animals.

### 4.3. Electroporation

For electroporation, the square wave pulse generator developed in Vilnius Gediminas Technical University (Vilnius, Lithuania) was used [44], which was further improved to support 100 ns–1 ms electric pulses up to 4 kV, 60 A. The pulses were delivered using adjustable parallel plate stainless steel electrodes (a 3 mm gap was used in the experiments). Two protocols were employed: PEF1: 12 kV/cm × 200 ns × 500 (0.006 J/pulse) and PEF2: 12 kV/cm × 500 ns × 500 (0.015 J/pulse), which were generated at 100 Hz. The protocols were designed to trigger saturated permeabilization rates, but different rates of tissue ablation (for comparison purposes), however, both cases feature no thermal effects (low pulse energy). In the case of calcium electroporation, a single injection of 168 mM CaCl_2_ in 0.9% NaCl solution was delivered into the tumors (approximately half of the tumor volume). The mice were treated with PEF after 5 min post injection of calcium.

### 4.4. Experimental Scheme

The mice were divided into seven groups. Three mice groups were controls: tumor-free mice (*n* = 5) – those mice were used as age mashed controls for immunological investigations; CTRL (*n* = 13) – tumor-bearing controls; CTRL + Ca (*n* = 5) – tumor-bearing mice with a single injection of CaCl_2_ into the tumors. The tumors of two mice groups (PEF1 (*n* = 9) and PEF2 (*n* = 10)) were treated with pulsed electric fields using the PEF1 and PEF2 protocols, respectively. The tumors of the last two groups of mice (PEF1 + Ca and PEF2 + Ca) were treated with calcium electroporation with a respective PEF protocol. Before the experimental procedures (day 0) the backs of mice were shaved, depilated using 8% Na_2_S aqueous solution and then rinsed with water. The mice were anesthetized by intraperitoneally injection of ketamine (80 mg/kg) and xylazine (10 mg/kg).

### 4.5. Evaluation of Tumor Sizes

Tumor sizes were evaluated by volumetric measurement and by luminescence of the tumors. During the volumetric measurement, the area of the scabs (formed after PEF treatment) and the volume of tumor were measured by digital caliper every 2–3 days. The area (mm^2^) of the scabs was calculated according the formula of ellipse: A = π × a/2 × b/2, where π = 3.1419, a, b—centered length and width of the scab. Tumor volume (mm^3^) was calculated according the formula: V = πlw^2^/6, where l—length and w—width of the tumor.

The luminescence of the tumors was imaged using IVIS Spectrum equipment (Caliper/Perkin Elmer, Akron, OH, USA) and Living Image Software Perkin Elmer, Akron, OH, USA). The mice were imaged prior to the treatment and immediately after the treatment with CaCl_2_ and PEF, three days after the treatment and in the time-points as indicated in the Results section. Prior to the imaging, the mice were injected intraperitoneally with 150 µL (30 mg/mL in PBS) of D-luciferin solution (Promega, Madison, WI, USA). After 10–15 min, animals were imaged under anesthesia. A mixture of 3% isoflurane (Vetpharma Animal Health, S.L., Barcelona Spain) in oxygen was used for introductory anesthesia and 1.5% mixture was used for maintenance anesthesia for the imaging in following days. The bioluminescence was proportional to the number of living SP2/0 Luc cells. Luminescence was expressed as photons/sec/region of interest (ROI) minus background luminescence of the same size region.

### 4.6. Flow Cytometric Analysis

Spleens, lymph nodes and pieces of tumors were mashed through a cell strainer into a 3.5 cm Petri dish with RPMI medium. Cells were centrifuged at 300× *g* for 5 min at room temperature. Lymph node and tumor cells were resuspended in a small amount of buffer for flow cytometry (2% fetal calf serum (FCS) and 0.1% NaN_3_ in phosphate buffered saline (PBS)). Splenocytes were resuspended in 15 mL of 0.16 M NH_4_Cl to lyse erythrocytes and incubated for 5 min before centrifugation. The centrifuged cells were resuspended in FACS buffer. Cell surface staining was performed by incubation of 0.3–0.5 × 10^6^ of cells in 20 µL of FACS buffer with anti-CD16/32 (Fc block) and mixture of requisite antibodies on ice for 30 min. The following stainings were performed: CD28-PE/CD62L-APC/CD44-fluorescein isothiocyanate in combination with CD4-PerCP or CD8-PerCP (for splenocytes, lymph node and tumor cells); Gr1-FITC/Mac1-APC/CD31-PE (for splenocytes); CD4-PE-Cy5/CD8-FITC/CD25-APC/Dx5-PE (for splenocytes) and CD4-PE-Cy5/CD8-FITC/CTLA4–PE-CF594 (for splenocytes). The antibodies used were obtained from BD Biosciences (San Jose, CA, USA); CTLA4–PE-CF594, CD31-PE, CD28-PE, CD44-FITC, CD4-PerCP, CD8-PerCP), Milteyi Biotec (Bergisch Gladbach, Germany); Gr1-FITC, CD8-FITC, Dx5-PE, Mac1-APC, CD62L-APC); BioLegend (San Diego, CA, USA); CD4-PE-Cy5) and eBioscience (San Diego, CA, USA); CD25-APC). The measurement and analysis were performed with aBD FACScalibur instrument (BD Biosciences, San Jose, CA, USA) and CellQuest software or with a FlowSight cytometer (Amnis Millipore, Burlington, MA, USA) and IDEAS software (Millipore, Burlington, MA, USA). The gating and analysis strategy is presented in the Supplementary Data, Figures S2–4.

### 4.7. Cytotoxic T Lymphocyte (CTL) Killing Assay

SP2/0 Luc myeloma cells (1 × 10^6^/mL in RPMI 1640 medium) were treated with 10 µg/mL of mitomycin C (Sigma-Aldrich, St. Louis, MO, USA) for 1.5 h at 37 °C and washed with RPMI medium. 10 × 10^6^ splenocytes and 0.2 × 10^6^ mitomycin treated SP2/0 Luc cells were plated in to one well of 24 well plate and co-cultured in RPMI medium supplemented with 2 mM glutamine, 100 U/mL penicillin, 100 mg/mL streptomycin, 10% FCS, 0.1 mM 2-β-mercaptoethanol and 10 ng/mL (10–25 U/mL) recombinant mouse IL-2 (Gibco, Thermo Fisher Scientific, Waltham, MA, USA) for 5 days. The cells were split as required at days 2 or 3. After a 5-day culture, the dead cells were removed by Lympholite M (Cedarline, St, Surrey, BC, Canada) gradient centrifugation. The effector cells were washed twice with medium without serum, and resuspended in RPMI 1640 medium supplemented with 2 mM glutamine, 100 U/mL penicillin, 100 mg/mL streptomycin, 10% FCS at a concentration of 20 × 10^6^ cells/mL. Seven 3-fold serial dilutions with a starting 20 × 10^5^ cells/well (V = 100 µL) were prepared in the wells of a 96-roundbottom well plate. SP2/0 Luc cells were used as target cells. 100 µL of target cells (2 × 10^5^ cells/mL) were distributed into the wells with effector cells. The controls were target cells alone. Replicates of three wells for each effector cell concentration were prepared. The plates were shortly centrifuged (1 min at 2500 rpm) and incubated for 6 h in humidified 37 °C, 5% CO_2_ incubator. In vitro bioluminescent assay was performed as described above. The results were expressed as ratio of effector/target cells when killing of tumor cells is 50%.

### 4.8. Determination of Tumor Cell-Specific Antibodies

Antibodies to surface and intracellular tumor cells antigens were determined. For this live and fixed/permeabilized SP2/0 Luc cells were used. SP2/0 Luc cells were fixed with 4% paraformaldehyde in PBS buffer for at least 10 min at room temperature, washed with PBS and permeabilized with 0.1% ice-cold Triton X-100 in PBS for 10 min. The cells were washed and resuspended in FACS buffer. 0.3 million SP2/0 Luc cells were used for one staining. The cells were incubated with diluted murine serum. The cells were washed with FACS buffer and incubated with antibodies to mouse immunoglobulins diluted in FACS buffer. The following antibodies to mouse immunoglobulins were used: goat a-mouse IgG FITC (Thermo Fisher Scientific, Waltham, MA, USA), anti-mouse IgM FITC (BD Pharmingen, San Jose, CA, USA), the mixture of biotin labeled antibodies to mouse IgG1, IgG2a, IgG2b, IgG3 (BD Pharmingen) with subsequent incubation with Avidin-FITC (BD Pharmingen). For elimination of dead/apoptotic cells the live SP2/0 cells were stained additionally with 7AAD (BD Pharmingen). The cells were measured with an Amnis Millipore FlowSight cytometer (Burlington, MA, USA) and analyzed with the IDEAS software (Millipore, Burlington, MA, USA). The negative control was cells stained with secondary antibodies only.

### 4.9. Statistical Analysis

All the data was analyzed using GraphPad Prism 6 software (GraphPad Software Inc., La Jolla, San Jose, CA, USA). Survival of the mice was analyzed with Kaplan–Meier Survival Analysis (Log-rank Mantel-Cox and Gehan-Breslow-Wilcoxon tests). A nonparametric Mann-Whitney U test was used to compare two groups of mice. Differences with a *p*-value < 0.05 were regarded as significant.

## 5. Conclusions

We have analyzed the efficacy of irreversible electroporation with calcium electroporation against SP2/0 tumors in vivo. The treatment based on IRE was effective against primary tumors, destroyed the tumor microenvironment and induced an anti-tumor immune response, however, it was not sufficient for complete control of tumor metastasis. The treated mice had enlarged spleens and increased spleen T cell numbers, lower percentages of suppressor cell subsets (conventional CD4^+^CD25^+^ Treg, CD4^+^CD25^−^DX5^+^ Tr1, CD8^+^DX5^+^, CD4^+^CD28^−^, CD8^+^CD28^−^), changed proportions of Tcm and Tef/Tem T cells in the spleen and increased amount of tumor cell specific antibodies in the sera. Higher energy treatment induced better antitumor efficiency, however, application of a series of treatments or combination with other methods during the first week is recommended to increase the number of complete responses.

## Figures and Tables

**Table 1:** Metastasis of SP2/0 Luc cells after electroporation of the tumors.

Mouse Groups		CTRL	CTRL + Ca	PEF1	PEF1 + Ca	PEF2	PEF2 + Ca
**Mice, total numbers**		13	5	9	8	10	10
Primary tumor did not renew	numbers	0	0	0	1	5	4
	% from total	0	0	0	12.5	50	40
Enlarged lymph nodes due to metastasis	numbers	3	2	5	7	7	4
	% from total	23.4	40	55.6	87.5	70	40
Fully recovered mice	numbers	0	0	0	1	1	2
	% from total	0	0	0	12.5	10	20

CTRL—tumor bearing control mice without treatment; CTRL+Ca—tumor bearing mice treated with CaCl_2_; PEF1 and PEF2—tumor bearing mice treated with PEF1: 12 kV/cm × 200 ns × 500 (0.006 J/pulse) or PEF2—12 kV/cm × 500 ns × 500. PEF1 + Ca and PEF2 + Ca—tumor-bearing mice treated with PEF and CaCl_2_.

## Supplementary Materials

The following are available online at https://www.mdpi.com/2072-6694/11/11/1763/s1, Figure S1: Flow cytometric analysis of CTLA expression on CD4 and CD8 T cells in spleen; Figure S2: Gating strategy of CD28-negative and Tem/Tef and Tcm CD4 and CD8 T cells; Figure S3: Gating strategy of myeloid suppressor Mac1^+^Gr1^+^CD31^+^ cells; Figure S4: Gating strategy of CD4^+^CD25^−^Dx5^+^ (Tr1), CD4^+^CD25^+^, CD8^+^Dx5^+^, CD4^+^CTLA^+^, CD8^+^CTLA^+^ and NK cells; Table S1: Expression of CD28 on Tem/Tef and Tcm T cells.
