# Supplementary material for: Antitumor Response and Immunomodulatory Effects of Sub-Microsecond Irreversible Electroporation and Its Combination with Calcium Electroporation
Source: Cancers (Basel). 2019 Nov 9;11(11):1763. doi: 10.3390/cancers11111763 (PMC6896087; doi:10.3390/cancers11111763)
Supplement: Supplementary file 1 [file cancers-11-01763-s001.pdf]

Supplementary Materials

Antitumor Response and Immunomodulatory Effects of Sub-Microsecond Irreversible Electroporation and Its Combination with Calcium Electroporation

Vitalij Novickij, Robertas Česna, Emilija Perminaitė, Auksė Zinkevičienė, Dainius Characiejus, Jurij Novickij, Saulius Šatkauskas, Paulius Ruzgys and Irutė Girkontaitė

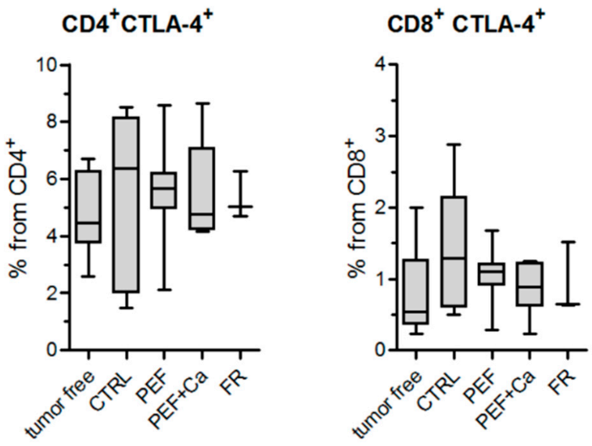

Figure S1. Flow cytometric analysis of CTLA expression on CD4 and CD8 T cells in spleen. Tumor free mice, CRTL (untreated and only CaCl2 treated mice), PEF – PEF2 treated mice, PEF+Ca- PEF2 and CaCl2 treated mice, FR (fully recovered). Gating strategy is presented in figure S3.

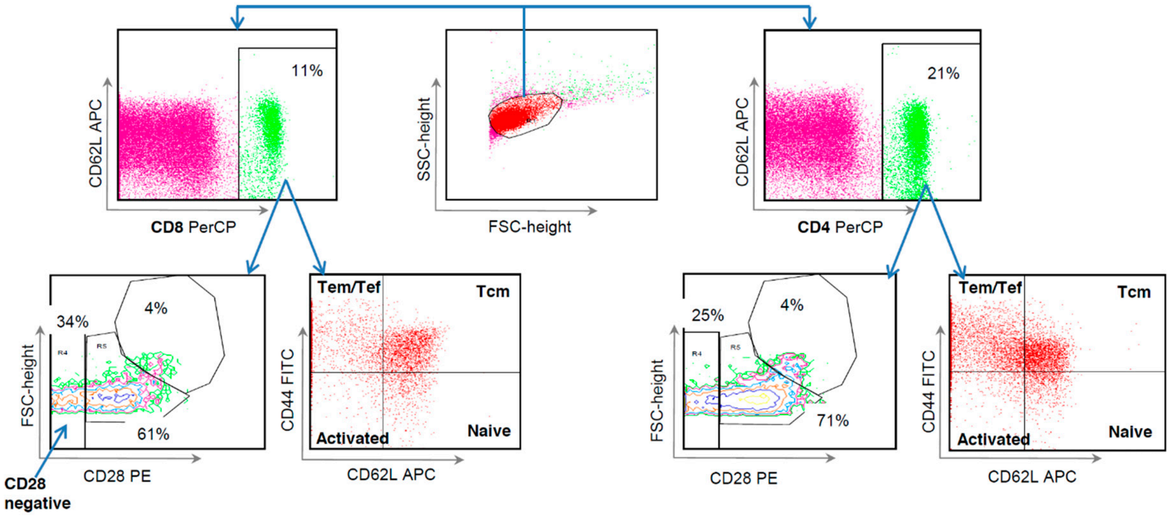

Figure S2. Gating strategy of CD28-negative, and Tem/Tef and Tcm CD4 and CD8 T cells. Splenocytes wre stained with CD28-PE/ CD62L-APC/CD44-FITC in combination with CD4-PerCP or CD8-PerCP and analyzed by BD FACSCalibur and CellQuest software.

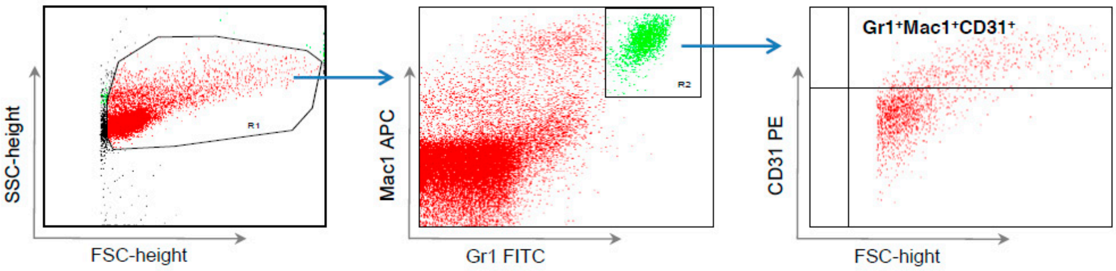

Figure S3. Gating strategy of myeloid suppressor Mac1+Gr1+CD31+ cells. Splenocytes were stained with Gr1-FITC/Mac1-APC/CD31-PE and analyzed by BD FACSCalibur and CellQuest software.

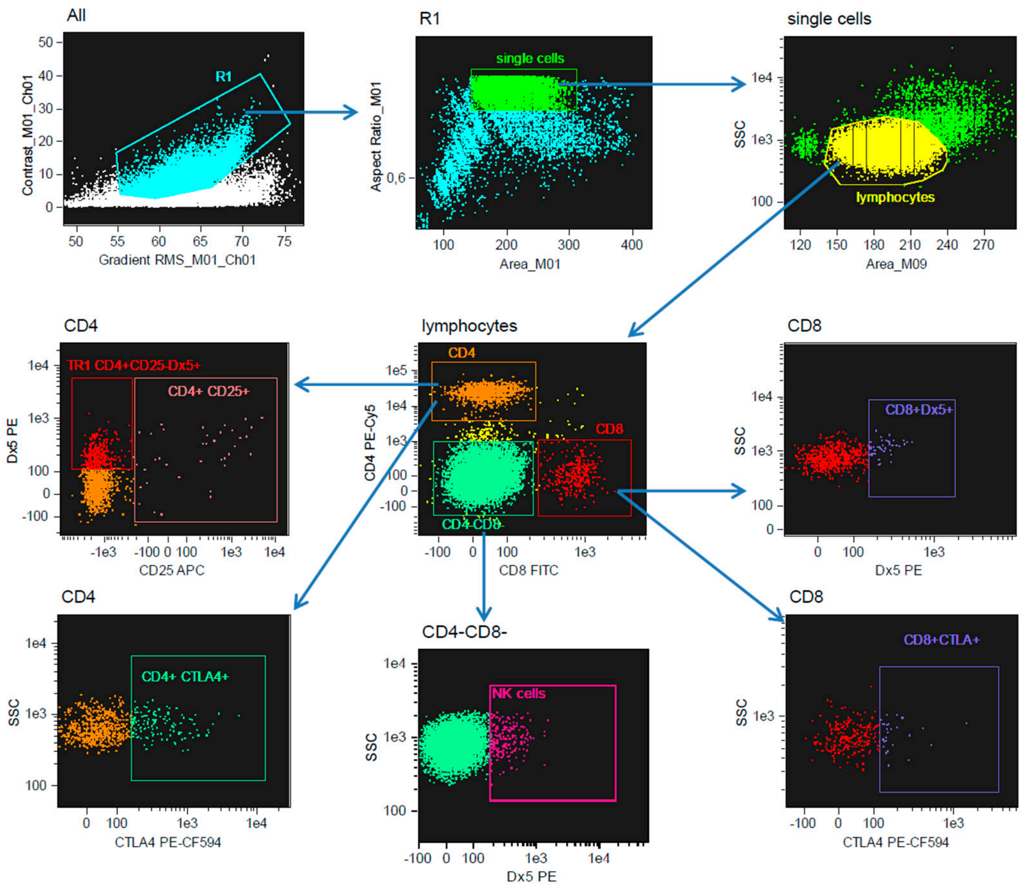

Figure S4. Gating strategy of CD4+CD25-Dx5+ (Tr1), CD4+CD25+, CD8+Dx5+, CD4+CTLA+, CD8+CTLA+ and NK cells. Splenocytes were stained with CD4-PE-Cy5/CD8-FITC/CD25-APC/Dx5-PE (for splenocytes) and CD4-PE-Cy5/CD8-FITC/CTLA4–PE-CF594 and analyzed by FlowSight (Amnis Millipore) cytometer and IDEAS software.

Table S1. Expression of CD28 on Tem/Tef and Tcm T cells.

	$CD28^{-/low}$	$CD28^{medium}$	$CD28^{high}$	$CD28^{-/low}$	$CD28^{medium}$	$CD28^{high}$	$CD28^{-/low}$	$CD28^{medium}$	$CD28^{high}$	$CD28^{-/low}$	$CD28^{medium}$	$CD28^{high}$
	% from $CD4^+$ $CD44^+CD62L^-$ (CD4 Tem/Tef)			% from CD4+ $CD44^+CD62L^+$ (CD4 Tcm)			% from $CD8^+$ $CD44^+CD62L^-$ (CD8 Tem/Tef)			% from CD8+ $CD44^+CD62L^+$ (CD8 Tcm)		
**Spleen**												
CTRL wt	25,5 ±2,8	64,7 ±4,7	7,6 ±1,9	20,9 ±4,2	53,3 ±7,1	23,4 ±5,7	15,9 ±5,4	56,9 ±6,2	24,6 ±9,9	31,1 ±5,3	56,7 ±3,9	12,4 ±3,5
CTRL	24,9 ±3,0	68,5 ±5,0	6,1 ±1,9	17,1 ±5,9	52,5 ±3,2	27,9 ±6,2	14,2 ±2,2	58,9 ±5,3	23,2 ±2,9	30,2 ±11,9	51,8 ±3,3	17,0 ±12,1
PEF2	22,8 ±3,1	**60,2 ±5,2**	**10,9 ±3,8**	19,4 ±3,4	49,8 ±2,9	27,2 ±3,5	14,8 ±5,4	52,6 ±7,6	29,3 ±11,0	32,8 ±3,6	56,2 ±4,6	10,9 ±2,8
PEF2+Ca	22,7 ±4,6	60,1 ±4,5	**12,0 ±6,4**	18,0 ±3,0	40,7 ±15,8	30,6 ±6,0	16,4 ±5,9	55,0 ±6,9	26,0 ±10,6	35,6 ±6,9	51,3 ±5,9	12,8 ±4,3
**Lymph nodes**												
CTRLwt	37,1 ±6,3	57,8 ±3,6	4,4 ±2,3	43,2 ±9,9	51,7 ±7,2	5,0 ±3,4	34,1 ±5,9	61,0 ±4,6	4,7 ±2,2	42,8 ±6,1	56,9 ±5,5	1,2 ±0,7
CTRL	35,1 ±8,5	62,4 ±7,4	2,6 ±1,6	39,6 ±14,8	57,7 ±14,4	3,4 ±1,7	39,7 ±14,5	57,2 ±11,6	3,3 ±2,3	45,8 ±15,3	54,3 ±14,0	1,2 ±1,4
PEF2	31,1 ±9,5	58,0 ±7,8	9,3 ±11,1	**27,5 ±11,9**	55,0 ±15,3	15,7 ±21,9	36,1 ±4,7	59,2 ±4,1	4,5 ±4,2	39,0 ±16,7	50,6 ±13,3	8,7 ±20,8
PEF2+Ca	32,1 ±3,7	60,3 ±3,1	6,5 ±5,6	32,3 ±6,2	58,9 ±3,9	8,1 ±5,3	36,8 ±5,6	58,1 ±5,2	4,9 ±2,4	41,6 ±3,7	57,7 ±2,3	1,6 ±1,4
**Tumor infiltrated lymphocytes (TIL)**												
CTRL	24,8 ±2,9	71,3 ±3,0	4,2 ±3,2	28,4 ±14,6	59,1 ±15,6	11,5 ±8,0	32,6 ±4,6	66,4 ±5,0	1,9 ±1,5	31,3 ±1,4	53,1 ±12,0	15,6 ±11,4
PEF2	17,5 ±8,0	64,2 ±9,2	5,1 ±2,9	14,9 ±12,2	60,3 ±9,6	18,0 ±8,4	**13,9 ±13,4**	41,5 ±33,0	**39,4 ±42,7**	**12,6 ±12,7**	61,5 ±12,5	24,1 ±18,5
PEF2+Ca	21,6 ±6,1	**60,6 ±6,2**	7,2 ±5,8	**13,9 ±7,5**	56,0 ±13,1	20,0 ±11,1	16,6 ±15,6	45,9 ±21,8	**37,2 ±37,3**	**12,9 ±17,0**	49,3 ±10,8	35,6 ±22,4

Tef/Tem -T effector memory cells, Tcm - T central memory cells. Significant differences ($p<0.05$) between the mice groups according to Mann Whitney test are in bold, where red - significant versus CTRL wt; blue - significant versus CTRL. CTRL wt – untreated mice without tumors; CTRL – tumor bearing control mice without treatment; CTRL+Ca – tumor bearing mice treated with $CaCl_2$; PEF1 and PEF2 – tumor bearing mice treated with PEF2 protocol –12 kV/cm × 500 ns × 500. PEF2+Ca – tumor-bearing mice treated with PEF2 and $CaCl_2$.

cc BY

© 2019 by the authors. Licensee MDPI, Basel, Switzerland. This article is an open access article distributed under the terms and conditions of the Creative Commons Attribution (CC BY) license (http://creativecommons.org/licenses/by/4.0/).
